# The complete chloroplast genome of *Liparis nervosa* (Orchidaceae)

**DOI:** 10.1080/23802359.2019.1698343

**Published:** 2019-12-11

**Authors:** Qing-Hua Zhang, Xiao-Ting Wang, Shu-Zhao Zheng, Wei-Yao Zhu, Zhang Song

**Affiliations:** Department of Forest Protection, Forestry College of Fujian Agriculture and Forestry University, Fuzhou, China

**Keywords:** *Liparis nervosa*, chloroplast genome, Orchidaceae, phylogenetic analysis

## Abstract

*Liparis nervosa*, a terrestrial orchid was widely used as a traditional medicinal plant in China. In this study, we assembled the complete chloroplast genome of *L. nervosa* using Illumina sequencing data. The whole genome is 158,716 bp, contains a large single-copy region (LSC 86,010 bp), a small single-copy region (SSC 18,276 bp), and a pair of inverted repeats (IR 27,215 bp). The complete genome has 132 genes, including 77 protein-coding genes, 38 tRNA genes, and 8 rRNA genes. In addition, a maximum-likelihood phylogenetic analysis demonstrated that *L. nervosa* was most closely related to *Oberonia japonica*. This work provides a theoretical basis for the development of conservation strategies of *L. nervosa*.

*Liparis nervosa* is an herbaceous plant belonging to the Orchidaceae family (Wu and Raven, 2009). It is a terrestrial orchid widely distributed in the tropical and subtropical regions of the world. In China, *L. nervosa* was used as a traditional medicinal plant for detoxicating and hemostatic functions for a long history. Previous studies have shown that various nervogenic acid derivatives, pyrrolizidine alkaloids, and active phenanthrenes were isolated from *L. nervosa* and they have antihypertensive, hypolipidemic, pro-coagulant, and antitumor activity (Huang et al. 2013; Song et al. 2013; Liu et al. 2016; Lin et al. 2018). However, due to the artificial exploitation and the destruction of the habitat, *L. nervosa* has become an endangered plant species in the China Species Red List (Wang and Xie 2004). The complete chloroplast genome of *L. nervosa* was assembled and annotated in this paper. The research not only will provide a support for in-depth study of *L. nervosa*, but also is helpful to lay a theoretical basis for the development of conservation strategies.

The samples of *L. nervosa* were collected from Qinglong waterfall scenic area, Yongtai, Fujian province, China (25°45′22.37″N, 118°57′48.36″E). The specimens are kept in the Herbarium of Fujian Agriculture and Forestry University (specimen code FAFU01847).

The total genomic DNA was extracted from leaves using a modified CTAB method (Doyle and Doyle 1987) and sequenced by the BGISEQ-500 platform. The clean reads were used to assemble the complete chloroplast genome and then annotated with the chloroplast genome of *Bletilla ochracea* (GenBank accession NC_029483) as the reference sequences. The assembly and annotation methods are described in detail in Ai et al. (2019). Finally, we obtained a complete chloroplast genome of *L. nervosa* and submitted to GenBank with accession number MN641753.

The complete chloroplast genome of *L. nervosa* is 158,716 bp in length with the GC-content of the whole plastome 36.9%. It consists of a large single-copy (LSC) region, a small single-copy (SSC) region, and two inverted repeat (IR) regions of 86,010 bp, 18,276 bp, and 27,215 bp, respectively. The new sequence has a total of 132 genes, including 77 protein-coding genes, 38 tRNA genes, and 8 rRNA genes.

In order to confirm the phylogenetic position of *L. nervosa*, a phylogenetic analysis was performed based on 12 complete chloroplast genomes of Orchidaceae species, including *Dendrobium xichouense* (NC_035341), *D. fanjingshanense* (NC_035344), *D. primulinum* (NC_035321), *Oberonia japonica* (NC_035832), *Cymbidium sinense* (NC_021430), *C. ensifolium* (NC_028525), *Bletilla ochracea* (NC_029483), *Eulophia zollingeri* (NC_037212), *Pleione bulbocodioides* (NC_036342), *Epipactis mairei* (NC_030705), *Neottia acuminata* (NC_030709), and *Goodyera schlechtendaliana* (NC_029364). All the sequences were downloaded from NCBI GenBank and aligned using MAFFT v7.307 (Katoh and Standley 2013). The phylogenetic tree was constructed by using a maximum likelihood analysis of MEGA 6.0 (Tamura et al. 2013) with 1000 bootstrap replicates. The results indicated that *L. nervosa* was most closely related to *O. japonica* (Figure 1).

**Figure 1.**
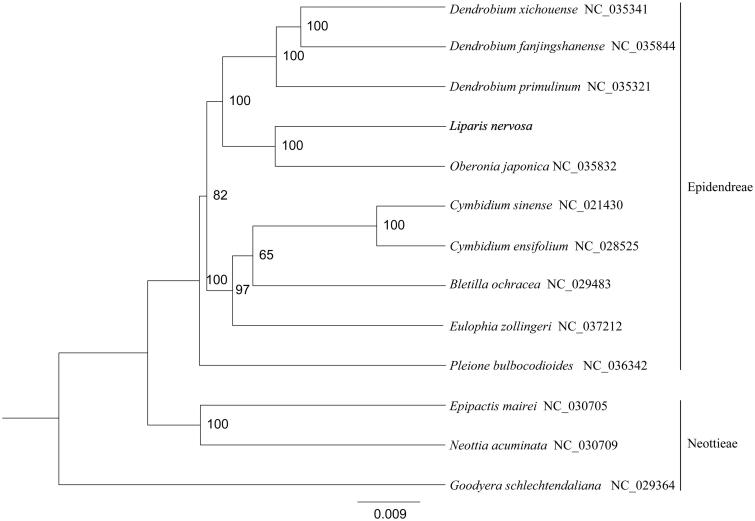
Phylogenetic tree constructed based on 12 complete chloroplast genome sequences of Orchidaceae. All the reference sequences were downloaded from NCBI GenBank.
